# A more principled use of the *p*-value? Not so fast: a critique of Colquhoun’s argument

**DOI:** 10.1098/rsos.181519

**Published:** 2019-05-15

**Authors:** Ognjen Arandjelović

**Affiliations:** School of Computer Science, University of St Andrews, St Andrews KY16 9SX, UK

**Keywords:** statistics, Bayesian, frequentist, empirical, evidence

## Abstract

The usefulness of the statistic known as the *p*-value, as a means of quantifying the strength of evidence for the presence of an effect from empirical data has long been questioned in the statistical community. In recent years, there has been a notable increase in the awareness of both fundamental and practical limitations of the statistic within the target research fields and especially biomedicine. In this article, I analyse the recently published article (Colquhoun 2017 *R. Soc. open sci.* **4**, 171085 (doi:10.1098/rsos.171085)) which, in summary, argues that with a better understanding and thus more appropriate use of the statistic, many of the aforementioned limitations can be addressed. In particular, I demonstrate that the (often implicit) premises of this counterargument are questionable, in some cases arguably inconsistent, and that therefore the counterargument provides little if any justification for the continued use of the *p*-value. Additionally, my analysis should help researchers seeking to interpret their empirical data by illustrating the nuanced nature and the multiplicity of statistical, methodological and epistemological issues which must be considered in this process.

## 1. Introduction

**Figure 1.**
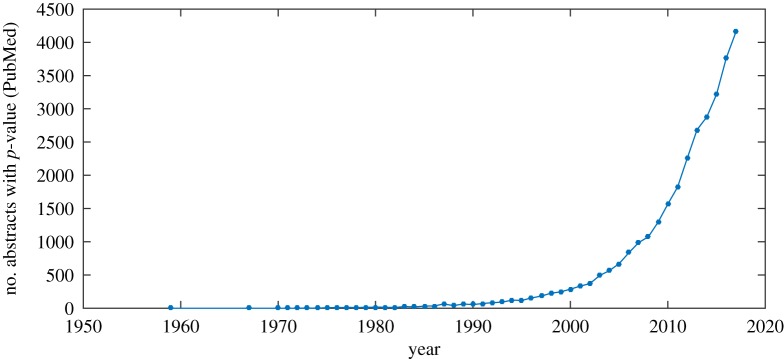
Number of abstracts per calendar year mentioning the *p*-value in articles indexed by PubMed.

It is no exaggeration to say that the use of the statistic commonly known as the *p*-value has become pervasive in the analysis of experimental evidence across a variety of research fields, as illustrated in figure 1. This trend is particularly evident in biomedical subjects [1]. Although criticisms thereof are not new [2], until recently they were largely confined to the niche of the statistical community. Due to the highly theoretical and mathematical nature of their underpinnings, these criticisms have for a long time struggled to penetrate into the ultimate target fields and be adopted by the researchers who ought to benefit the most. However, in no small part driven by alarming reproducibility concerns and statistical manipulations [3,4], recently there has been an increase in the awareness of a variety of concerns related to the *p*-value as a statistic itself [2,5], as well as the manner in which it is employed, interpreted and understood [3,6].

A recently published article authored by Colquhoun attempts to present a new look at how the *p*-value *is being* used vs. how it *should be* (in the author’s opinion) used [7]. Colquhoun presents a series of arguments, which conceptually can be summarized as being critical of the way the *p*-value is *understood and interpreted* (which I agree with), arguing in effect that with a change in the manner in which the statistic is used, the key concerns raised by an increasing number of researchers, can be addressed adequately.

Many of the points in Colquhoun’s article seem convincing at first sight. However, his case is underlain by a series of questionable implicit assumptions and inconsistencies, and insufficiently developed practical proposals. Many of these weaknesses are subtle, which highlights the need for them to be rebutted, as superficially they do seem to address comprehensively and with rigour the most important criticisms in the existing literature.

## 2. Language and hidden fallacies

The definition, the correct interpretation and the common misunderstandings of the *p*-value have all been addressed at length and in detail by numerous works in the existing literature (indeed, Colquhoun too presents a good overview [7]) so I shall refrain from rehashing them, and instead refer the reader to a number of selected articles [2,7] and move straight to the main topic of the present work.

Considering what is arguably the most foundational and philosophically pivotal question in the discussion of *p*-value, I would like to begin with the following statement from Colquhoun’s article:

It is not uncommon to hear the view that the point null hypothesis is not worth testing because it is never exactly true.

Right here I would like to state clearly that I agree with the author that this indeed is a commonly expressed view and one that I too support [8]. Colquhoun takes the argument head on and attempts to rebut it with the following counterargument:

Apart from the fact that it can be exactly true (just give the same pill to both groups) …

As I remarked earlier, this rebuttal seems solid. Yet, it is flawed: the *same pill* that I consume cannot be consumed by another individual. The cognitive trap that Colquhoun has fallen into is a result of the lack of rigour of everyday language. This lack of rigour itself should not be surprising considering the forces which have for most of its existence driven the development of colloquial speech. I understand that this criticism may come across as frivolous semantic pedantry of no practical consequence: ‘of course what the author *meant* to say was “pills with the same contents”, as everybody would have understood’. Yet, this argument not only does not rebut the criticism but in fact *strengthens* it, for it highlights the additional layer of uncertainties and a lack of perfect control of a pill’s contents. If a pill is supposed to contain an active ingredient, the actual amount of it is subject to variation governed by the manufacturing process. The same is true of placebo pills which do not contain the ingredient of primary interest, or indeed any other form of intervention or treatment. And, lest it be forgotten, there is always the possibility of the presence of factors which affect the outcome and are not known, and which by this very fact were not controlled for (in everyday speech, these have become known as ‘unknown unknowns’) [9].

It is interesting and insightful to add here that uncertainties such as those highlighted above, fit into and are effortlessly incorporated within Bayesian approaches, which I do not think would be unfair to say Colquhoun at times treats rather dismissively. For example, latent variables and the manufacturing process statistics can be used to include in the modelling the associated probability density functions which can be integrated over in the course of the inference process.

## 3. What about priors?

In an attempt to justify the fundamental premises the use of the *p*-value is based upon, Colquhoun states that:

… the prior distribution for the null hypothesis is a spike located at zero effect size.

and adds:

This seems to me to be an entirely reasonable approach.

There are several aspects of this argument which are rather unsatisfactory. Most obviously, there is little to be found in terms of an attempt at justifying the claimed reasonableness of the aforementioned assumption. Surely, both qualitatively (i.e. where the peak is located) and quantitatively (i.e. how peaked the prior is) should depend on our understanding of the underlying phenomenon which is being examined.

Moreover, the assumption as expressed above is vague and lacking in clarity. What precisely does ‘a spike’ mean? Surely, this spike cannot be in the form of the Dirac *δ* function as this would imply a strict *impossibility* of any effect being present. But what is the functional form of the suggested prior then? There are an infinite number of functions which peak at zero, many of which are frequently used as priors in statistics. This question relates to yet another unclear aspect in Colquhoun’s argument. In particular, I am referring to the repeated references to the desire to assess:

… whether our experiment is consistent with a true effect size of zero.

Considering the argument which I have already introduced, that based upon the fact that stochastic variability is practically unavoidable, every experimental outcome is consistent with the effect size of zero, in the sense that it is a possible outcome. An experiment comprising 1000 coin tosses and resulting in 1000 heads up is entirely consistent with the hypothesis of fair coin tosses (the probability *p* = 1/2^1000^ is small but nevertheless positive). Here again, we come to the issue at the crux of the problem: that the wrong question is being asked. As noted by many statisticians before, the question is not that of consistency with a single hypothesis but of the probability density distribution across the entire space of possible hypotheses [2] and, if desired, selecting the ‘best’ of these (for a related discussion the reader would be well advised to consult Ng’s contributions to the debate [10]).

In addition to the above, while Colquhoun is happy to claim the reasonableness of the argument that ‘the prior distribution for the null hypothesis is a spike located at zero effect size’ he flips the proverbial burden of justification in the subsequent criticisms of the Bayesian methodology, now claiming that:

… the prior distribution …will never be known anyway.

Here too are hidden a series of subtle presumptions. A detailed analysis of these would require more space than suitable here and is therefore out of the scope of the present article, but a few are worth pointing out. Firstly, the author’s use of the word ‘known’ and the consequent binarization into ‘known’ and ‘not known’ is inappropriate on a basic epistemological level given that we are effectively dealing with an inductive inferential process. Moreover, the statement assumes that there is *the correct* and true prior. This is a much debated issue in the Bayesian community and many (Subjective Bayesians as opposed to Objective Bayesians) consider this view as ill-conceived, instead regarding priors as expressing subjective belief (possibly informed and constrained by the symmetry of the problem, etc.) [11]. And, of course, there is always the possibility of choosing an uninformed prior [12].

Relating both to the fallacy just discussed, as well as that which was the subject of the previous section, is the following claim by Colquhoun:

Recently, it was asserted that if we observe a *p*-value just below 0.05, then there is a chance of at least 26% that your result is a false positive [2].

As should be clear from my argumentation thus far, this claim fundamentally does not make sense— given that there are an infinite number of hypotheses consistent with the observed data (in that they *could* have generated the data), no single hypothesis (including the null) can have a finite probability associated with it. We always need to be talking about probability density functions and ranges of hypotheses (e.g. probability that the effect is smaller than *x*). Even the choice of words is strikingly singular. In particular, I am referring to the word ‘asserted’. The claim in the quote is not one that can be meaningfully asserted but should be proven, or demonstrated (with the same meaning as ‘proven’).

Lastly, while I do not think it necessary to elaborate on this further, I do feel compelled to draw the reader’s attention to Colquhoun’s statement that:

This [*prior, peaked at zero effect*] is, of course, what statisticians have been teaching for almost a century.

It should be clear that this is a textbook case of the *argumentum ad antiquitatem* fallacy (with a fair dose of needless *argumentum ad verecundiam*) which carries little to no weight in technical debate in which all parties are informed about the subject discussed.

## 4. Segmentation of the analytical pipeline

Now I would like to take a step back and consider a subtle and often overlooked issue which concerns the entirety of the analytical process within which Colquhoun’s proposed approach for the use of the *p*-value is employed. It can be summarized well by the following sentence from the original article:

If we decide that our results are not consistent with a true effect size of zero then we can go ahead and calculate our estimate of the effect size.

Putting aside my already detailed criticisms of the fundamental premise of the approach, that is that the question of consistency of evidence with the effect size of zero is an ill-conceived one, I would like to consider the consequences of separating the inference of ‘if there is an effect’ (step 1) and of ‘if likely present, how large the effect is’ (step 2).

Much like before, Colquhoun leaves the question of how the second step is to be performed without much elaboration, presumably as something that is ‘straightforward’. Yet, when the entirety of the argument in the article is considered, the proposed approach is far from clear. Connecting this with the content of §3, would Colquhoun advocate the use of a frequentist methodology or a Bayesian one? In either case, why not adopt this approach from the very outset and focus on the inference of the best hypothesis (here I feel compelled to refer the reader to Ng’s work [10]) without singling out the null and unnecessarily breaking up the analytical process? As I argued before, this approach which I termed ‘atomization’ can lead to the loss of important information [9]. The principled and clinically more relevant approach would start by seeking the answer to the question of what the probability of at least a certain effect size (governed by clinical needs) is.

## 5. Summary and conclusion

The widespread use, especially in biomedical research, of the statistic known as the *p*-value as a means of assessing the strength of evidence for the presence of an effect of interest has been attracting an increasing amount of criticism in recent years. In the present article, I analysed a recent proposal by Colquhoun on how the statistic could be applied in a more principled manner, which, in summary, argued that with its better understanding and thus more appropriate use, many of the aforementioned criticisms can be addressed. Though I challenge a number of his ideas, I welcome Colquhoun’s contribution as it provides a good basis for illustrating the nuanced nature and the multiplicity of statistical, methodological and epistemological issues which must be considered in the analysis of empirical data. I believe that my analysis has demonstrated comprehensively that the criticisms of the *p*-value are well founded and theoretically solid, strengthening the case against the continued use of the statistic even with the reasonable safeguards described by Colquhoun.

## Data accessibility

Not applicable; the present article addresses methodological issues and does not use or refer to any specific datasets.
